# Brain Mapping-Aided SupraTotal Resection (SpTR) of Brain Tumors: The Role of Brain Connectivity

**DOI:** 10.3389/fonc.2021.645854

**Published:** 2021-03-02

**Authors:** Giuseppe Roberto Giammalva, Lara Brunasso, Roberta Costanzo, Federica Paolini, Giuseppe Emmanuele Umana, Gianluca Scalia, Cesare Gagliardo, Rosa Maria Gerardi, Luigi Basile, Francesca Graziano, Carlo Gulì, Domenico Messina, Maria Angela Pino, Paola Feraco, Silvana Tumbiolo, Massimo Midiri, Domenico Gerardo Iacopino, Rosario Maugeri

**Affiliations:** ^1^Unit of Neurosurgery, Department of Biomedicine, Neuroscience and Advanced Diagnostics, Post Graduate Residency Program in Neurosurgery, University of Palermo, Palermo, Italy; ^2^Department of Neurosurgery, Cannizzaro Hospital, Catania, Italy; ^3^Department of Neurosurgery, ARNAS Garibaldi, Catania, Italy; ^4^Section of Radiological Sciences, Department of Biomedicine, Neuroscience and Advanced Diagnostics, University of Palermo, Palermo, Italy; ^5^Neuroradiology Unit, S. Chiara Hospital, Trento, Italy; ^6^Department of Neurosurgery, Villa Sofia Hospital, Palermo, Italy

**Keywords:** supratotal resection, brain mapping, connectomics, brain connectome, high-grade gliomas, low-grade gliomas, brain tumor, extent of resection

## Abstract

Brain gliomas require a deep knowledge of their effects on brain connectivity. Understanding the complex relationship between tumor and functional brain is the preliminary and fundamental step for the subsequent surgery. The extent of resection (EOR) is an independent variable of surgical effectiveness and it correlates with the overall survival. Until now, great efforts have been made to achieve gross total resection (GTR) as the standard of care of brain tumor patients. However, high and low-grade gliomas have an infiltrative behavior and peritumoral white matter is often infiltrated by tumoral cells. According to these evidences, many efforts have been made to push the boundary of the resection beyond the contrast-enhanced lesion core on T1w MRI, in the so called supratotal resection (SpTR). SpTR is aimed to maximize the extent of resection and thus the overall survival. SpTR of primary brain tumors is a feasible technique and its safety is improved by intraoperative neuromonitoring and advanced neuroimaging. Only transient cognitive impairments have been reported in SpTR patients compared to GTR patients. Moreover, SpTR is related to a longer overall and progression-free survival along with preserving neuro-cognitive functions and quality of life.

## Introduction

Gliomas constitute a common type of primary brain tumor. Malignant histological subtypes (high-grade gliomas, HGGs), are classified by the World Health Organization (WHO) as either grade III or IV tumors. Glioblastoma (GBM) is the most common and aggressive malignant primary brain tumor (1–7). It carries an unfavorable prognosis with a median overall survival of 12–18 months and early death after diagnosis in case of no intervention (8–10). Currently, surgical intervention represents the first stage of GBM therapy and it is well documented that the extent of surgery has the key role in affecting the patient overall survival (OS) (8, 10, 11).

Contrast-enhancement on brain MRI is commonly considered a consequence of the blood-brain barrier (BBB) permeabilization because of the tumor infiltrative behaviour; consequently, the boundaries of contrast-enhancement on brain MRI are considered to reflect the margins of the tumoral lesion (10). In support of this assumption, the extent of resection (EOR) of the tumoral lesion is independently correlated to survival time. In facts, it has been reported that the resection of more than 95% of the contrast-enhancement mass, or residual tumor volume lower than 2 cm^3^ are independently associated with improved OS and delayed recurrence (12). According to these evidences, EOR has been assumed as a metric to judge the success of tumor resection and to predict improved long-term outcomes, such as progression-free survival (PFS) and OS (13): in particular, lesser residual tumor volume is directly correlated to longer OS (10).

Low grade gliomas (LGGs - WHO grade II) are less frequent than HGGs; they are usually diagnosed in young adults with no or mild neurological and neuropsychological impairments and they are characterized by a better prognosis (12). Their growth is characterized by gradual and slow infiltrating behaviour through the adjacent brain tissue; The slow-growing pattern of LGGs induces brain plasticity phenomena which may result in functional compensation and may explain the lack of detectable neurological impairments in LGGs (14).

A significant correlation between the EOR and the OS in LGGs has been demonstrated by MRI-based volumetric studies (12). Even if with lesser extent than HGGs, LGGs infiltrate the adjacent normal-appearing brain parenchyma and tumor cells have been found up to 20 mm beyond the area of MRI pathological boundaries. According to this evidence, LGGs are considered potential malignant tumors since the diagnosis, thus early and aggressive surgical treatment is advised (12, 15). Currently, the main purpose of LGGs treatment is to delay the malignant transformation by reaching the supratotal resection of normal-appearing but infiltrated brain parenchyma, in order to increase patient OS and to preserve quality of life (QoL) (12, 16). It has been clearly demonstrated that supratotal resection positively influences the natural history of LGGs compared to the only GTR which has been associated more frequently to malignant transformation (12). Considering the easier access to brain imaging and consequent earlier diagnosis, more patients are discovered with incidentally and asymptomatic LGGs. In these cases, preventive surgery may be considered legitimate for the lower morbidity related to surgery, the higher rate of successful supratotal resection and the strong impact on the OS (12, 15, 17–21).

Nowadays, GBM is considered not only a highly proliferative tumor with high rate of recurrence even after radical surgery (8), but it should be also considered as a “diffuse disease of the brain” migrating along the white matter tracts (18). In fact, it was histologically demonstrated that tumoral cells may be found far from the primary lesion, beyond the enhanced boundaries on T1-weighted brain MRI (22, 23). According to these evidences, MRI imaging may underestimate the real extent of the tumor (24); thus, a Gross Total Resection (GTR, defined as the removal of the T1-weighted contrast-enhanced zone on brain MRI) of the tumor may not be enough (13). Despite EOR up to 100% of the contrast-enhanced tumor volume (10), it has been shown that tumoral infiltration may be found within 2 to 3 cm from the border of the original lesion (8), making tumor recurrences inevitable and mostly located near the resection cavity (10). Consequently, the infiltrative nature of GBM cells makes it difficult to eliminate microscopic disease and macroscopic GTR should not be considered a complete resection (8). In this setting, research has gone so far to broaden the contribution of gliomas surgery extending the concept of just a “tumorectomy” (18). The concept of supramarginal resection has been developed to describe the resection of the peritumoral tissue beyond the distinctive enhanced tumor mass on T1-wheighet brain MRI, with the aim to remove the microscopically infiltrated surrounding brain parenchyma (10, 11).

## Materials and Methods

An extensive systematic literature review was performed according to PRISMA guidelines on PubMed, MEDLINE and Scopus databases using the following keywords: “supratotal resection”, “supramarginal resection” “supratotal resection AND GTR” “supratotal resection AND connectomics”, “supratotal resection AND brain mapping”, “supratotal resection AND brain connectivity”, “supratotal resection AND glioma”, “brain mapping AND glioma”. Meta-analyses, review, clinical series and case reports were included. Non-English works and studies lacking of full text were excluded. After the initial identification, each article was screened according to the topic of this review and only articles discussing the feasibility and application of SpTR in brain gliomas were selected. Moreover, pre- and intra-operative brain mapping techniques were enlightened in order to clarify their application in case of SpTR and their relationship with brain connectomics. Among the selected articles, we included those concerning the concept of connetome and brain connectomics, the role of SpTR for the treatment of brain gliomas and the pre-operative and intra-operative tools and techniques aimed to perform SpTR.

## Results

**Figure 1:**
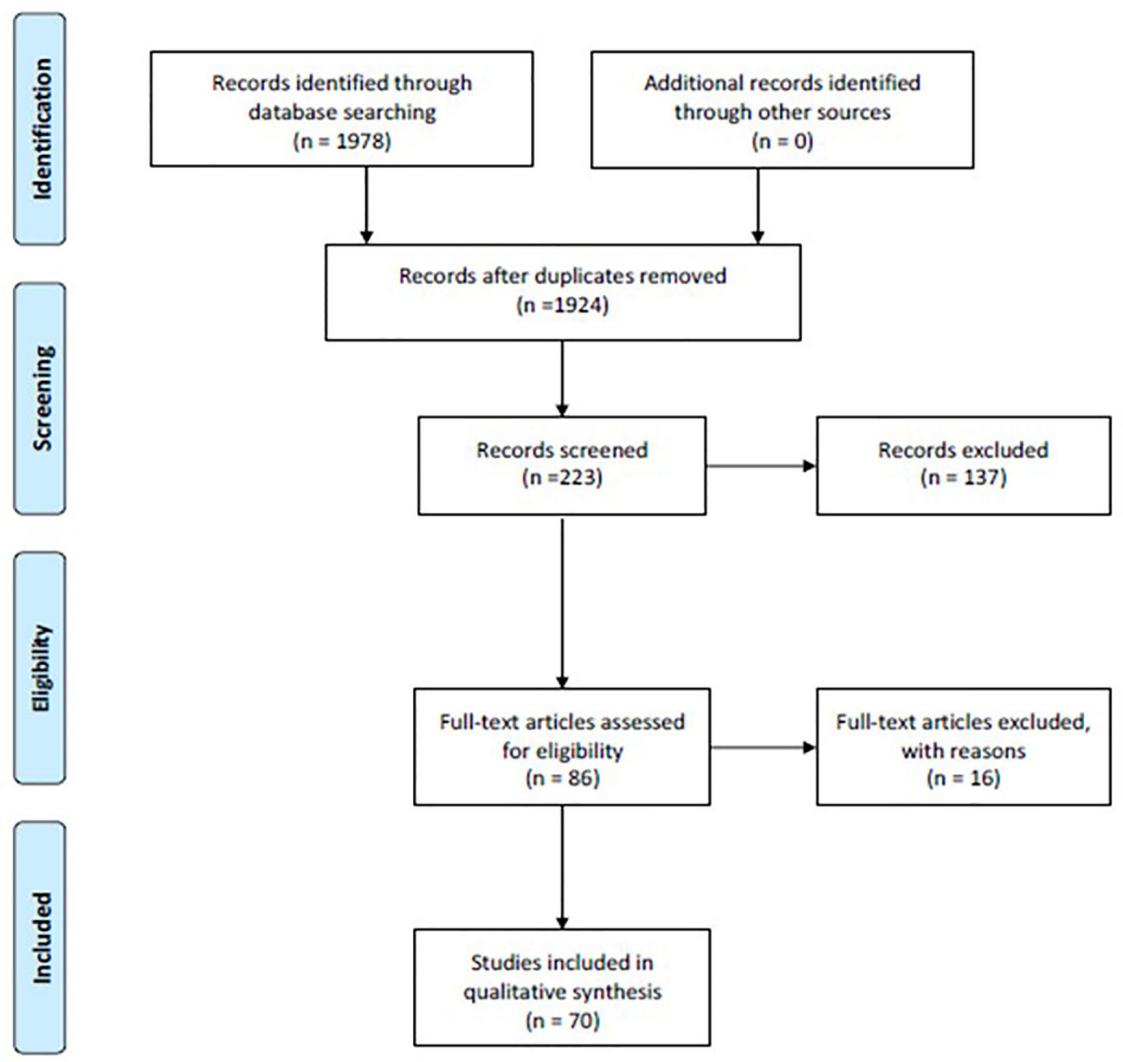
Flow-diagram of the results of this systematic review according to PRISMA statement.

Through a careful analysis of the literature, we obtained an insightful review of the current applications of brain mapping-aided SpTR for brain gliomas. From the first queries, 1924 unique records were identified. These records were screened according to our above mentioned inclusion criteria; thus, 223 articles were identified and 137 articles were later excluded due to the lack of full text or relevance according to the topic of this review and our inclusion criteria. From the 86 full-text articles assessed for eligibility, 16 more articles were excluded because of the lack of relevance about brain mapping and connectomics in SpTR. Finally, after a careful revision, we included in this systematic review 70 articles (**Figure 1**).

## Discussion

### Brain Connectomics and SupraTotal Resection (SpTR) of Brain Gliomas

During the last decade, the principle of locationism and the distinction of eloquent areas have been replaced by the emerging concept of brain “connectomics” (25). Connectomics is a novel multidisciplinary paradigm in which the brain is seen as a complex network of individual components interacting through continuous communication (26). This paradigm overcomes the former existence of eloquent areas and it relies on the concept of “connectome”, which represents the interconnection of every part of the brain through white matter fibers (27).

According to this concept, focal and slow growth tumors determine an upsetting of normal functional relations within the brain, leading to an anatomical reshaping and functional reconfiguration of both cortical and subcortical networks even far beyond the tumor borders (26). Thus, the functional sequelae of brain tumors should be considered also at a global level since these functional changes influence whole-brain functional complexity and network architecture (26, 28). Moreover, the effects of tumor removal are related to the individual network robustness since the removal of the surrounding peritumoral brain might be functionally counterbalanced and might not result in significant additional functional impairment (26). This opens up the doors to the concept of onco-functional balance. According to this, surgical resection is guided by pre-operative assessment and intraoperative functional mapping and it takes account of the considerable structural-functional variability and the individual neural dynamics across glioma patients, in order to significantly increase patient’s OS and to decrease the rate of neurological impairments and the consequent disability (17, 29). This rising concept paves the way even for the treatment of tumors within brain structures previously considered inoperable (17).

The adoption of supratotal resection (SpTR) is a further step toward the treatment of brain gliomas. SpTR was firstly adopted for the treatment of diffuse LGGs, taking advantage from the reshaping of brain networks induced by neuroplasticity and related to LGGs slower growth rate; then, SpTR has been also adopted for the treatment of HGGs (12, 30).

It has been shown that the complete resection of T1w contrast enhanced tumoral tissue together with the resection of more than 53,21% of the surrounding T2-weighted fluid-attenuated inversion recovery (FLAIR) abnormalities is associated whit a longer OS, than in case of less extensive resection (12, 31). The concept of SpTR is not limited to the T1-w contrast enhanced volume as boundaries of tumor resection but it tends toward a maximal resection of the T2/FLAIR signal abnormalities (18). Obviously, an extensive tumor resection could dramatically increase the risk of neurological deterioration and negative influence on patient OS (10). Thus, actual preservation of neurological function and maintenance of QoL are being actively pursued as a fundamental aspect of treating patients with gliomas, and improvement in QoL is considered to be an integral part for determining OS (1). This reveal a fundamental change from the idea of a surgery related to anatomical boundaries to the idea of a surgery guided by functional boundaries (18). The aim of this new surgical concept is to safely push the boundaries of surgical resection through FLAIR abnormalities under the guidance of functional brain-mapping until eloquent structures have been encountered. This type of functional guidance relies on an accurate study of the individual brain functional anatomy, intraoperative mapping through cortical and sub-cortical electrical stimulation, neurophysiological monitoring, and intraoperative imaging guidance (32, 33). This underlines the importance of safest surgical resection possible according not only to anatomical but also to functional boundaries. Since the OS is directly related to the EOR, SpTR may be taken into account not only in case of involvement of non-eloquent structures but also when a mild neurological impairment with few consequences on daily life is an acceptable price to pay for the patient in order to prolong OS while attending a neurological recover (18).

### Pre-Operative Evaluation, Functional Imaging, and Cognitive Assessment

A careful preoperative selection of patients who can benefit from surgery is mandatory in order to meet the most favorable onco-functional balance (17).

Pre-operative imaging technique is accomplished MRI using different and specific sequences (34). Functional MRI (fMRI) plays a key role for the identification of motor cortex and language dominance through the evaluation of regional blood flow (BOLD or blood-oxygen-level dependent) changes, thus contributing to the analysis of connectome (35). Two different types of fMRI could be performed: task-based fMRI and task-free resting state fMRI. Since the execution of repetitive tasks could create artifacts in the evaluation of BOLD signals, resting-state fMRI is considered a more reliable mapping technique in pre-operative surgical planning (36). However, fMRI reliability is influenced by perfusion changes induced by different gliomas (37), in particular in HGGs (38).

Diffusion Tensor Imaging (DTI) and Diffusion Tensor Tractography (DTT) represent two MRI techniques which are capable to depict subcortical white matter tracts. DTI relies on evaluation of diffusion tensor basing on diffusion indexes of water molecules. Starting from DTI, DTT is employed to depict subcortical neural networks basing on the orientation of axonal bundles according to their anisotropy; this technique shows the capability to depict white matter tracts through the direction of water molecules, to identify signal anomalies and axonal integrity (39). Unfortunately, DTI is prone to distortions during computation of fiber-tracking algorithm (40). However, DTT is a useful preoperative tool for tridimensional representation of the fiber tracts capable to influence the surgical strategy; moreover, more complex DTI processing and the corroboration of intraoperative monitoring such ash Direct Electrical Stimulation (DES) may overcome DTI limitations thus allowing a precise functional evaluation and estimation of EOR in glioma patients (39, 40).

Recently, functional neuroimaging (fMRI, DTI, DTT) has been being supported by navigated transcranial magnetic stimulation (nTMS). Through nTMS it is possible to accurately map eloquent and motor areas using magnetic stimulation. nTMS can significantly reduce surgical time and guarantee a better functional outcome if coupled intraoperatively with DES (41). fMRI, nTMS, and DES guarantee a continuous control of motor, sensory or language domains on awake patients, thus ensuring a more radical excision according to functional boundaries (42).

Besides functional imaging, neuropsychological assessment plays a key-role in the pre-operative functional evaluation. Through the administration of several different tasks it is possible to gather information about patients’ cognitive pre-operative status and to diagnose functional impairments about information processing speed, attention, working memory, verbal memory, visual memory, executive and phasic functions (43, 44). In order to perform a standardized neuropsychological assessment several tests have been developed and validated to explore several neurological domains; some of them can also be run on a friendly and common device such an iPad (45–50).

### Intraoperative Monitoring and Surgical Technique During Brain Mapping-Guided SpTR

Onco-functional balance represents the crucial node of SpTR in order to obtain the maximal feasible resection without unrecoverable functional impairments (12). For this purpose, a novel surgical perspective which relies on integrated preoperative and intraoperative functional evaluation is demanded (12, 30, 51).

Feasibility of SpTR is related to some functional and technical issues. Firstly, surgical resection extended beyond contrast-enhanced tumoral margins on T1-w MRI could interfere with the functionality of neighbouring eloquent areas. Secondly, infiltrated brain tissue with low density of tumoral cells could not be correctly discriminated by normal brain tissue, leading to partial or non-complete resection (10, 52, 53). In order to achieve SpTR while preserving neurological functions, image-guided surgery must be overtaken and replaced by a functional-guided surgery (12).

The most reliable intraoperative method to directly identify functional neural networks is intraoperative DES during awake surgery. DES uses a biphasic electrical current to generate direct transient stimulation or interference within cortical or subcortical networks (54). DES could be associated by intraoperative sensorimotor localization, which relies on phase reversal technique (PRT) and it is capable to localize the transition between sensitive and motor cortex through the registration of somatosensory evoked potential (SSEP) electrical phase (54).

Usually, intraoperative functional mapping through electrostimulation is performed during staged “asleep-awake-asleep” surgery (55). In the first stage, cortical areas are exposed and local markers are placed along the tumor borders before surgical manipulation. Functional areas are detected through neuronavigation on former functional brain imaging and through DES. During the second stage, the patient is awakened. Basing on preoperative functional assessment, patient undergoes selective tasks related to the tumor localization; during tasks execution, surgeon simultaneously apply DES on peritumoral areas in order to evoke incorrect or inappropriate neurological response if functional network is stimulated (25, 45). During this stage, DES is alternated to tumor excision in order to identify functional boundaries which will limit the resection; then, the patient is asleep in order to perform haemostasis and closure (55, 56). During the whole procedure, somatosensory and motor evoked potentials are recorded and continuous electrocorticograms is performed to detect discharge phenomena during direct brain stimulation and tumor resection (45).

This surgical technique allows surgeon to remove non-functional areas within a functional “security boundary” and to obtain greater EOR without an increased risk of permanent neurological impairments (12, 55). According to this evidence, tumor resection may be extended even to eloquent networks in order to optimize the “onco-functional” balance (57).

Brain mapping techniques have demonstrated increased rates of SpTR and a subsequent increased OS, especially in LGGs (22, 25, 32, 54, 55, 58). Intraoperative DES in awake patients allows a dramatic decrease in permanent neurological impairment, while increasing transient ones which are mostly recoverable (54, 55). On the other hand, surgical strategy for resection of HGGs should be more tailored on an accurate balance between EOR and preservation of cognitive functions since the short time for neurological recovery before the mandatory postoperative treatments (radio- and chemo-therapy) (16, 59).

Anatomical boundaries of gliomas during surgical resection may be enlightened by the use of fluorophores such as 5- aminolevulinic acid (5-ALA) and sodium fluorescein, intraoperative MRI (iMRI) and intraoperative ultrasound (IOUS) in order to verify the extent of resection (10, 30, 51, 60–66). iMRI with integrated functional neuronavigation is commonly used to achieve better visualization of residual tumor volume and to reassess neuronavigation during surgical manipulation to overcome brain shift (60, 67). As an adjunct, IOUS with our without contrast enhancement (CEUS) is a valuable tools to distinguish tumoral tissue from normal brain parenchyma; notably, IOUS is more accessible than iMRI and it immediately allows a real-time visualization of tumoral tissue during surgical manipulation (68). As regards fluorescent dyes, 5-ALA is specifically accumulated by glioma cells and it is enlightened by intraoperative source of blue-light (10, 52, 53, 69). The use of fluorescent dyes with neuronavigation guarantees greater EOR than the only neuronavigation. The maximum rate of resection could be achieved by combining fluorescent dyes and neuronavigation into the “dual intraoperative visualization approach” (DiVA), which permits further improvement in EOR and a consequent prolonged OS (10, 30, 51, 61, 62, 70).

## Conclusions

Despite extended surgical resection, LGGs and HGGs are still burdened by the possibility of tumor recurrence. Specific selection criteria are needed before surgery in order to achieve the best possible result in removing safely the maximum of infiltrated brain tissue beyond tumoral margins. SpTR represents a novel concept of glioma surgery which relies on the evaluation of brain connectomics. SpTR reflects the effort to reach the best oncological outcome while preventing any permanent neurological and/or cognitive postoperative impairment, thus preserving patient’s QoL and accordingly increasing OS.

## Author Contributions

Conceptualization: RM, GG, LaB, RC, and FP. Methodology: RM, MP, and GS. Validation: LuB, GU, and PF. Formal analysis: FG and CeG. Investigation: LaB and RC. Data curation: GG, PF, and CaG. Writing—original draft preparation: GG, LaB, RC, and FP. Writing—review and editing: RG and DM. Supervision: RM and DI. Project administration: ST, MM, and DI. All authors contributed to the article and approved the submitted version.

## Conflict of Interest

The authors declare that the research was conducted in the absence of any commercial or financial relationships that could be construed as a potential conflict of interest.
